# Correlation between clarithromycin resistance, virulence factors and clinical characteristics of the disease in *Helicobacter pylori* infected patients in Shahrekord, Southwest Iran

**DOI:** 10.1186/s13568-021-01310-9

**Published:** 2021-11-03

**Authors:** Razieh Sadat Hosseini, Ghorbanali Rahimian, Mohammad Hadi Shafigh, Majid Validi, Mansoor Khaledi, Abolfazl Gholipour

**Affiliations:** 1 Cellular and Molecular Research Center, Basic Health Sciences Institute, Shahrekord University of Medical Sciences, Shahrekord, Iran; 2 Department of Internal Medicine, Shahrekord University of Medical Sciences, Shahrekord, Iran; 3 Department of Microbiology, Faculty of Medicine, Shahed University, Tehran, Iran; 4 Clinical Biochemistry Research Center, Basic Health Sciences Institute, Shahrekord University of Medical Sciences, Shahrekord, Iran

**Keywords:** *Helicobacter pylori*, Antibiotic resistance, Real-time PCR, Clarithromycin, Virulence factors

## Abstract

The purpose of this study was to determine the mutations associated with clarithromycin resistance in *Helicobacter pylori* strains isolated from biopsy samples that were collected from the endoscopic ward of Shahrekord Hajar teaching Hospital and also to study the frequency of virulence factor and their correlation and pathological findings with clarithromycin resistance during the years 2019–2020. In this cross-sectional descriptive study, 152 patients with *Helicobacter pylori* infection were considered, and then, two common A2142G and A2143G mutations in the 23SrRNA gene associated with resistance were analyzed by Real-time PCR (Taq man). The presence of vacA, iceA1, iceA2, cagA, babA2, and oipA virulence genes was investigated by PCR and electrophoresis in 8% polyacrylamide gel. Then, data were analyzed using the relevant statistical tests. In this study, the frequency of *Helicobacter pylori* was 76% and the frequency of mutant isolates was 57.2%. The frequencies of A2142G and A2143G point mutations were 42.1% and 28.3%. There was a significant correlation among oipA, vacA, and iceA1 virulence factors, type of disease, chronic inflammatory score, and glandular atrophy with the antibiotic resistance to clarithromycin. There was no significant correlation between the age and sex of the patients with antibiotic resistance. According to the results of this study, it seems that the use of clarithromycin to combat this bacterium should be limited.

## Key points

1. Correlation between Clarithromycin resistance, Virulence factors and clinical Characteristics of the disease in *Helicobacter pylori* infected patients in Shahrekord, Iran, have novelty.
2. There is a significant relationship between clarithromycin resistance and oipA, VacA and the type of disease is reported for the first time in Iran.
3. Based on our knowledge, a significant association between glandular atrophy and degree of inflammation with resistance to clarithromycin has been reported for the first time in this study in the world.
4. There are already known facts about the topics under discussion and they are stated in "Discussion" section.

## Introduction

*Helicobacter pylori* is a gram-negative, microaerophilic, and spiral bacterium identified in 1982 (Marshall Warren 1984). *Helicobacter pylori* is recognized as the cause of gastritis, gastrointestinal ulcers and gastric carcinoma (gastric, intestinal ulcers), MALT lymphoma, and non-gastric diseases associated with *Helicobacter pylori* (Azadegan-Dehkordi et al. 2020; Fujikawa et al. 2003; Khaledi et al. 2020; Lee et al. 2005; Sanaii et al. 2019). The outcome of infection with this bacterium depends on several factors such as bacterial isolates, host inflammatory responses, host genetic diversity, and environmental factors (Montecucco de Bernard 2003; Nahid-Samiei et al. 2020; Rhead et al. 2007; Sanaei et al. 2020).

The prevalence of *Helicobacter pylori* varies worldwide (Thung et al. 2016). 50% of the population developed countries and 80% of the population developing countries are infected by this bacterium (Hooi et al. 2017; Khademi et al. 2015). As for the prevalence of the bacterium and its related diseases, proper treatment is very important. The antibiotic resistance of this bacterium is progressively increasing and has become a global concern and is also an important factor in determining the outcome of treatment. Standard treatment is a three-stage drug that consists of one acid neutralizer and two antibiotics, clarithromycin, and amoxicillin or metronidazole for 14 days (Sugano et al. 2015). Unfortunately, nowadays, the success of this treatment is less than 80% worldwide (Zhang 2015). Unsuccessful treatment includes a variety of causes including lifestyle habits such as smoking, bacterial isolates, immunodeficiency, antibiotic resistance, and inadequate treatment (Malekzadeh et al. 2009; Sanches et al. 2016). Unfortunately, the resistance of *Helicobacter pylori* to these two antibiotics in most of the countries of the world has led to the failure of the first line treatment to become a Problem (Ji et al. 2016). The most effective treatment regimens in Iranian studies have been recognized to be quadruple regimens based on clarithromycin or furazolidone (Malekzadeh et al. 1997; Roghani et al. 1999). Clarithromycin is a family of macrolides having a bacteriostatic effect, which binds to the peptidyl transferase 23SrRNA subunit of the large subunit of the bacterial ribosome and inhibits the protein-making process (Sanches et al. 2016). Also, clarithromycin resistance is the result of structural changes in this region. These changes decrease the tendency of the binding of clarithromycin to the target site of the peptidyl transferase ribosomes of the bacterium, and consequently, cause lack of protein inhibition and the main reason for these structural changes is the point mutations in the 23SrRNA region. In fact, resistance to clarithromycin is due to a spontaneous mutation in the chromosome in the V domain of the 23SrRNA gene. Adenine and guanine displacements have been reported at different points and mostly at the points of A2143G and A2142G, and less at the point of A2142C (Ghotaslou et al. 2015; Malekzadeh et al. 2009). A bacterium is very heterogeneous and its virulence varies geographically. Virulence factors help bacteria pathogenic but may determine treatment outcome. Most of the virulence factors examined in *H. pylori* are *cagA* and vacA (Taneike et al. 2009). Studies have been conducted on the correlation between virulence factors and antibiotic resistance worldwide. Because there are contradictory reports from different parts of the world about the relationship between virulence factors and disease type and pathological findings with antibiotic resistance. Also, there has been no study in Iran on the relationship between the presence of virulence factors iopA, vacA, babA2 iceA, and the type of disease and pathological findings with antibiotic resistance. This study aimed to investigate the relationship between resistance to clarithromycin with virulence factors of iceA, vacA, cagA, babA2, oipA, disease type, and pathological findings in Chaharmahal-o- Bakhtiari region of Iran. And based on our knowledge, a significant association between glandular atrophy and degree of inflammation with resistance to clarithromycin has been reported for the first time in this study.

## Materials and methods

### Study ethics

This study was approved by the Ethics Committee of Shahrekord University of Medical Sciences under the code IR.SKUMS.REC.1397.314. The patients included in the study were given the written consent and patient's information questionnaire.

### The community understudy

This study was performed on the symptomatic patients with *Helicobacter pylori* infection who were referred to endoscopy ward of Hajar Teaching Hospital from January 2019 to May 2020 in Chaharmahal and Bakhtiari Province.

### Inclusion criteria include

(1) Having the ability and willingness to participate in the study, (2) People over 15 years of age, (3) *Helicobacter pylori* infection was confirmed based on biochemical and molecular tests (PCR, RUT) and pathology. Also only the samples that were positive in all 3 methods were included in the study.

### Exclusion criteria included

(1) People taking aspirin or non-steroidal- anti-inflammatory drugs. (2) Those with metabolic disorders and immunosuppression. (3) People who have recently used antibiotics. (4) People younger than 15 years old and pregnant and lactating women were not included in this study.

### Sampling

200 people participated in the study. 3 biopsy specimens were received from the antrum of suspected patients that one of them was examined by rapid urease method, one specimen was pathologically examined and the other specimen was stored at − 70 °C. Then, the DNA was extracted by DNA extraction kit Bioflux (Japan) in terms of the kit instructions. Also, all the extracted DNA were kept in freeze at − 70 °C until use. *Helicobacter pylori* infection was confirmed based on biochemical and molecular tests (PCR, RUT) and pathology. Also only the samples that were positive in all 3 methods were included in the study. Among 200 patients, 152 were diagnosed with *Helicobacter pylori* infection.

### Molecular diagnosis

PCR test to amplify a 16SrRNA gene fragment to confirm the presence of *Helicobacter pylori* were performed using primers, temperature conditions, and concentration and volume of the proposed material by De Francesco et al. (2010) using Astec thermocycler (Japan) on all biopsies. Then, the amplified fragment was visualized using 8% polyacrylamide gel and stained with silver nitrate. Based on the results of PCR molecular test, RUT test and pathology, 152 samples were confirmed for *Helicobacter pylori*. These samples were also analyzed by PCR assay for the presence of *iceA1*, *iceA2*, *vacA*, *cagA*, *babA2*, *and oipA* genes using the primers, temperature, concentration, and volume of the materials proposed by Bagheri et al. including 35 cycles of denaturation (at 94 °C for 30 s), annealing (at 58 °C for 30 s, extension at 72 °C for 30 s), and one final extension (at 72 °C for 5 min). To investigate susceptible and resistant isolates of two common A2142G and A2143G mutations in the 23SrRNA gene related to clarithromycin resistance, Real-Time PCR (Taq man) and primer pairs and probes, temperature, and concentration of De Francesco et al. proposed materials were used. Real-Time-PCR reactions were performed in a total volume of 25 μl containing 3 μl of synthesized DNA solution, 12.5 μl of 2 × Rotor-Gene Probe PCR Master Mix (Ampliqon, Denmark), 1 μl DMSO, 500 nM of each primer, and 250 nM of the TaqMan probe (TAG Copenhagen, Denmark). Amplification program included a pre warming step (5 min at 95 °C), denaturation step (95 °C for 20 s), and an annealing (58 °C for 20 s)/extension step (72 °C for 20 s) (Bagheri et al. 2013), that were performed in Corbett (Australia).

All reactions were repeated three times and each time positive and negative controls were tested. Also, each mutation was separately examined. All reactions were repeated thrice and each time positive and negative controls were tested. In addition, each mutation was separately examined. Due to the lack of access to standard isolates, several clinical isolates were sequenced.

### Statistical analysis

Statistical analysis was performed using SPSS-18 software for Windows (IBM SPSS statistics, version 16.0.0; SPSS, Chicago, IL, USA). Chi-square test, fisher exact test and Logistic regression was used to investigate the association between patient and isolate characteristics in *Helicobacter pylori* clinical isolates with mutations related to clarithromycin resistance. P-value less than 0.05 was considered significant.

## Results

### Resistance to clarithromycin

The clinical and demographic characteristics of the patients are presented in Table 1. Among 200 patients, 152 were diagnosed with *Helicobacter pylori* infection. That the prevalence rate of *Helicobacter pylori* is 76%, among them the frequency of the isolates with mutations was 57.2% and the percentage of isolates without mutation was 42.8%. In general, the percentage of A2142G1 point mutation was 42.1%, of which 3.3% had homozygous genotype and 38.8% had heterozygous genotype. Also, the percentage of A2143G point mutation is 28.3% that 7.9% of the isolates had homozygous genotype and 20.4% of the isolates had heterozygous genotype.Twenty (13.2%) isolates showed both A2142G and A2143G mutations. The most frequent 2142G mutation was 42.1%.

**Table 1:** Clinical and demographic characteristics of the patients

Variable	N (%)
Gender	
Male	74 (48/7)
Female	78 (51/3)
Age group	
Under 30	11 (7/2)
30–60	98 (64/5)
Upper 60	43 (28/3)
Histology findings	
Chronic inflammatory	152 (100)
Acute inflammatory	123 (80/9)
Lymphoid follicle	25 (16/4)
Intestinal metaplasia	12 (7/9)
Glandular atrophy	74 (48/6)
Endoscopic findings	
Gastritis	90 (59/2)
Peptic ulcer	62 (40/8)

(Homozygous samples are samples that have mutations in two strands of DNA and Heterozygous samples are samples that have mutations in One strand of DNA).

### Virulence factor

In this study, the frequency of bacterial virulence genes were *cagA* 105 (69.1%),*oipA 77* (50.7%), *babA2* 92(60.5%), and *vacA*: s1 83 (54.6%), s2 21 (13.8%), s1s2 28 (18.4%), m1 45(29.6%), m2 62 (40.8%), m1m2 11(7.2%), s1m1 38 (25.0%), s1m2 32 (21.1%), s2m2 17 (11.2%), s2m1 2 (1.3%), *iceA1* 56 (36.8%), and *iceA2 78* (51.3%), respectively.

### Correlation between vacA, cagA, babA2, oipA, iceA1, and iceA2 virulence genes with resistance to clarithromycin

**Fig. 1:**
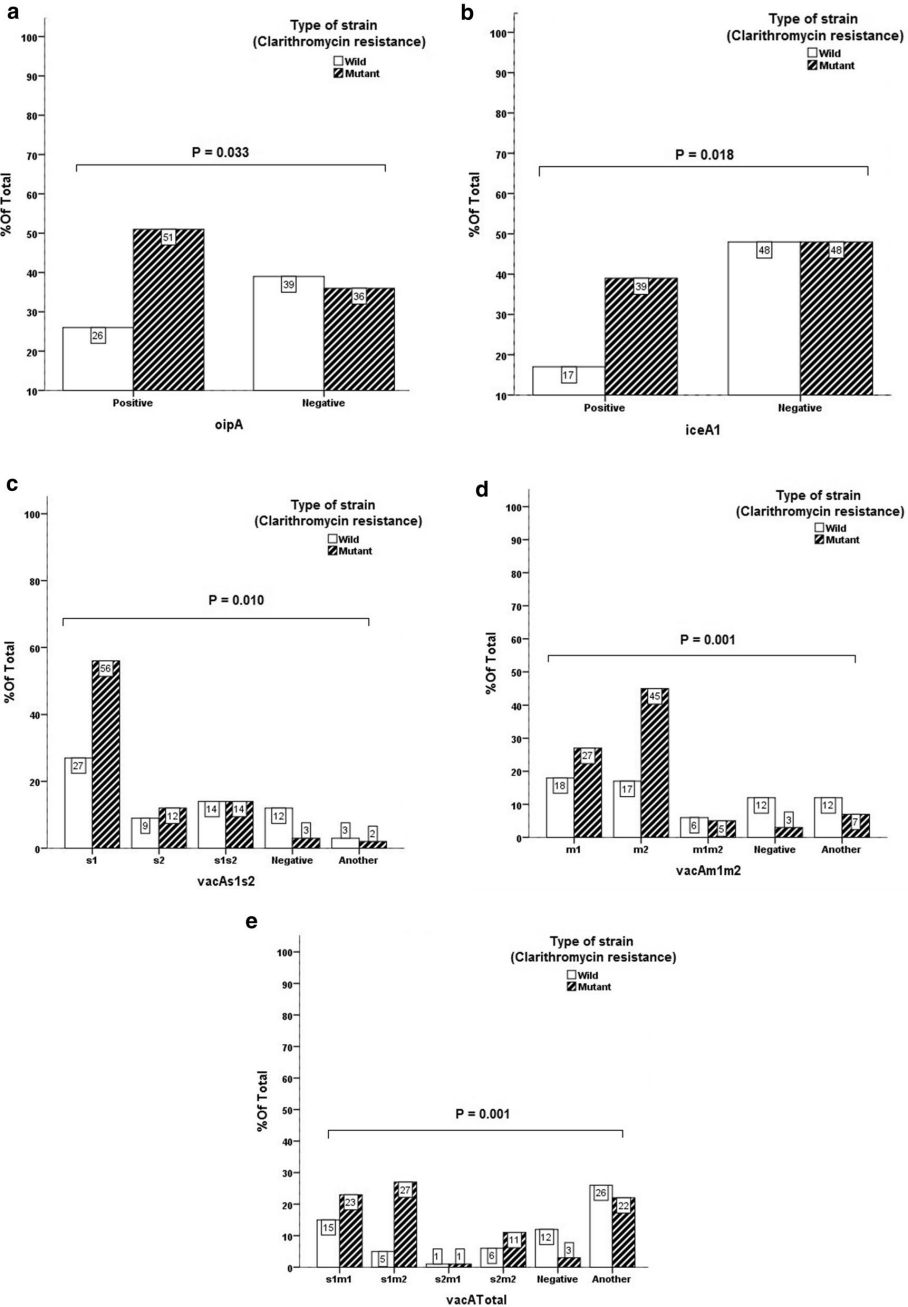
Correlation between the presence of virulence genes, *oipA* (**a**), *iceA1* (**b**), *vacAs1s2* (**c**), *vacAm1m2* (**d**), *vacATotal* (**e**) and antibiotic resistance

The correlation among the presence of virulence genes, *iceA1*, *iceA2 vacA*, *cagA*, *babA2*, and *oipA* in the studied isolates were investigated as well as the presence of point mutations related to clarithromycin resistance. There was a significant correlation among the presence of *oipA* (P-value = 0.033), the presence of *vacAm1m2*, *vacATotal* (P-value = 0.001) and *vacAs1s2* (P-value = 0.010), and the presence of *iceA1* (P-Value = 0.018) with antibiotic resistance. *vacATotal* in logistic regression test showed a significant relationship with clarithromycin resistance that the odds ratio for its different levels are 1.168, 5.763, 0.56, 1.671 and 0.312. Most isolates with virulence genes *oipA*, *vacA*, and *iceA1* have antibiotic resistance to clarithromycin, as shown in Fig. 1.

### Correlation between disease type and resistance to clarithromycin

**Fig. 2:**
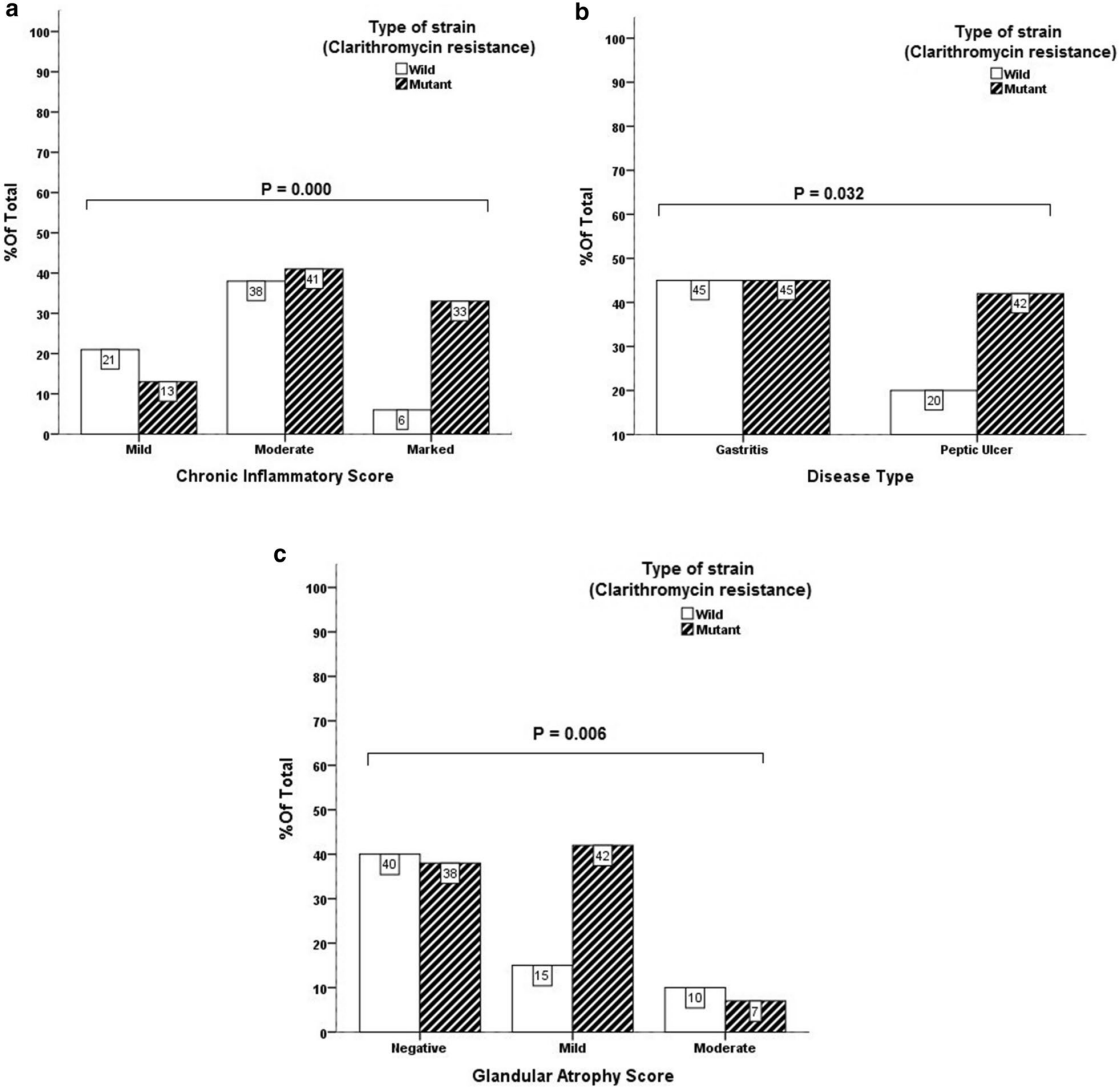
Correlation among the degree of inflammation (**a**), disease type (**b**), and Glandular atrophy (**c**) with the type of isolate in terms of clarithromycin resistance

There was a significant correlation between antibiotic resistance to clarithromycin and the type of disease, in people with gastritis (P-value = 0.032), which is considered as a mild form of *Helicobacter pylori* infection, in which *Helicobacter pylori* isolates are involved without antibiotic mutation. However, the isolates causing gastrointestinal ulcer are mostly resistant to clarithromycin antibiotics, as shown in Fig. 2.

### Correlation between age and gender by resistance to clarithromycin

Patients' age was categorized into three groups (under 30, 30–60, and over 60 years old). The association between that and the sex of the patients with antibiotic resistance was examined; however, no significant correlation was observed between them.

### Correlation between pathology findings and clarithromycin resistance

There was a significant correlation between clarithromycin resistance and a degree of chronic inflammation (P-value = 0.000). Accordingly, most of the people with mild chronic inflammation have a isolate *Helicobacter pylori* without mutation, and most of the people with moderate to severe inflammation have mutant isolates. Chronic inflammation in logistic regression test showed a significant correlation with clarithromycin resistance that the odds ratio for its different levels are 0.2 and 0.12. There was also a significant correlation between antibiotic resistance to clarithromycin and glandular atrophy (P-value = 0.006). Most people without glandular atrophy have wild-type or antibiotic-sensitive isolates, and those with glandular atrophy (mild to severe) often have mutant or clarithromycin-resistant isolates, as indicated in Fig. 2.

## Discussion

This study aimed to determine the mutations associated with clarithromycin resistance in *Helicobacter pylori* isolates, and also to investigate its correlation with virulence factors *iceA1*, *iceA2*, *vacA*, *cagA*, *babA2*, and *oipA* and the type of disease and pathological findings.

In this study, it was found that, clarithromycin resistance was significantly correlated with some virulence factors, type of disease, and degree of inflammatory. In the present study, the rate of clarithromycin resistance was 57.2% and the A2142G mutation was recognized as the most common mutation. According to the patients, they have not yet received antibiotic treatment for their infection, therefore should probably be considered as the actual primary resistance.

This resistance rate is higher than in southern European countries like Spain, (32.01%), and Portugal (42.35%), that they have the highest resistance to clarithromycin among European countries(Almeida et al. 2014; Molina–Infante et al. 2013). Level with some Asian countries such as Korea (60%), China (52%), India (58.8%) and some Northern European countries like Ireland (60.6%) and parts of the United States like Texas (50%) (Brennan et al. 2018; Mitui et al. 2014; Pandya et al. 2014; Thung et al. 2016). Also, according to a 2012 report by Kargar et al., which reported 35.98% of clarithromycin resistance in Chaharmahal and Bakhtiari Province (Kargar et al. 2011), it has been shown that the resistance to clarithromycin has increased 1.5-fold over the past 9 years. Clarithromycin resistance is due to the increased macrolide intake not only in *Helicobacter pylori* treatment, but also due to increasing in the treatment of respiratory tract infections. Also for this reason, rapid and accurate screening of clarithromycin-resistant isolates is clinically important. The failure of first-line treatment due to resistance to this drug has not only chronicled the disease and the increased its other side effects, but has also led to the increased financial burden worldwide and to the limited use of antibiotics in treatment (Goudarzi et al. 2016; Roghani et al. 1999). In this study, it was found that, there is a significant correlation among virulence factor *oipA* gene, *vacAm1m2,vacATotal, vacAs1s2*, and *iceA1* gene with antibiotic mutation in relation to clarithromycin (P = 0.033), (P = 0.001), (P = 0.010), and (P = 0.018). The majority of *vacA oipA*, and *iceA1* positive isolates have mutation and resistance to clarithromycin. In fact, this result confirms the correlation between resistance and the presence of virulence factor iceA1, *oipA*, and *vacA*, but no significant correlation was found between clarithromycin resistance and virulence factors *cagA and babA2*. Studies on the association between virulence factors and antibiotic resistance are very conflicting, So far, no studies have been conducted in Iran on the correlation between the presence of virulence factor *oipA*, *vacA*, *babA2*, and *iceA*, and antibiotic resistance. The association of these factors with antibiotic resistance was reported for the first time in this study. The current study data on the association of virulence genes *oipA*, *iceA*, *and vacA* with clarithromycin antibiotic resistance are primarily consistent with data from Treiber et al. (2002), Karabiber et al. (2014), and Boyanova et al. (2010), and subsequently, the correlation between the presence of virulence factor and antibiotic resistance regardless of the type of factor the studies support and endorse Khan et al. (2012), Brennan et al. (2018), and Wang et al. (2019). To justify this, it can be said that, isolates with virulence factors, especially *oipA*, produce inflammatory cytokines like IL-8 and cause aggressive disease like gastrointestinal ulcer. As a result, the patient has painful and uncomfortable symptoms, so starts taking antibiotic drugs that results in a failure in completing the course of treatment, and inadequate use and various other factors make the involved isolate resistant to antibiotics. The results of this study on virulence factors *babA2* and *cagA* showed no significant correlation between the presence of these factors and clarithromycin resistance. This is consistent with the results of studies by Wang et al. (2019), Godoy et al. (2003), Baglan et al. (2006), Broutet et al. (2001), and Lõivukene e al. (2000). In this study, there was no significant correlation between age and sex of the patients with clarithromycin resistance. This is in line with the results of Korona et al. (2019) and Elviss et al. (2004) and contrasts with the results of Wang et al. (2019), Chang et al. (2019), and Treiber et al. (2002). In this study, a significant relationship was observed between the degree of chronic inflammation and Glandular Atrophy with antibiotic resistance. Frequency of clarithromycin resistance in the patients with moderate to severe chronic inflammation and mild to moderate Glandular Atrophy were different compared with the patients with mild chronic inflammation and those without Glandular Atrophy, based on our knowledge, we are reporting this for the first time. Considering that between these two factors, chronic inflammation in both Chi-square and logistic regression tests showed a significant correlation with clarithromycin resistance, it is reported with a higher probability percentage, which indicates the importance of processing on this topic.

In this study, there was a significant correlation between antibiotic resistance to clarithromycin and type of disease in the patients infected by *Helicobacter pylori* (P = 0.032). The frequency of clarithromycin resistance was significantly higher in the patients with gastrointestinal ulcer compared to the patients with gastritis. This study is in line with the study of Treiber et al. (2002) and contrary to the results of Wang et al. (2019), which can be interpreted as isolates that cause an aggressive state of the disease like peptic ulcer. Because of painful and irreversible symptoms, the patient received bacterial and infection treatment. The patient has begun taking antibiotics, and failure to complete the course of treatment, inadequate use, and various other factors have made the isolates involved, resistant to antibiotics. This study was performed the first time in Iran investigating the correlation among clarithromycin antibiotic resistance and disease type and pathologic findings in *Helicobacter pylori,* and reported a significant correlation between these variables. Given these results, it is suggested that, the use of different from clarithromycin drugs and regular treatment in the patients with peptic ulcer may be much more necessary than before, because most of the isolates obtained from patients with peptic ulcer are resistant to clarithromycin, antimicrobial used in first-line treatments. These results indicate the necessity of determination the antibiotic resistance patterns of *H. pylori* before prescribing antibiotics, that this would help identify patients who are not suitable for clarithromycin-based therapies.

## Data Availability

The data are available. All data generated or analyzed during this study are included in this study.

## Abbreviations

PCR: Polymerase chain reaction
vacA: Vacuolating cytotoxin A
cagA: Cytotoxin associated genes A
oipA: Outer inflammatory protein A
RUT: Rapid urease test
DMSO: Dimethyl sulfoxide
MALT: Mucosa-associated lymphoid tissue

## References

[CR1] Almeida N, Romãozinho J, Donato M, Luxo C, Cardoso O, Cipriano M, Marinho C, Fernandes A, Calhau C, Sofia C (2014). *Helicobacter pylori* antimicrobial resistance rates in the central region of Portugal. Clin Microbiol Infect.

[CR2] Azadegan-Dehkordi F, Shirzad H, Ahmadi R, Bashash D, Abdollahpour-Alitappeh M, Luzza F, Larussa T, Samiei M, Rahimian G, Shafigh M-H, Bagheri N (2020). Increased Indoleamine 2, 3-Dioxygenase expression modulates Th1/Th17/Th22 and Treg pathway in humans with *Helicobacter Pylori*-Infected gastric mucosa. Hum Immunol.

[CR3] Bagheri N, Taghikhani A, Rahimian G, Salimzadeh L, Azadegan Dehkordi F, Zandi F, Chaleshtori MH, Rafieian-Kopaei M, Shirzad H (2013). Association between virulence factors of *Helicobacter pylori* and gastric mucosal interleukin-18 mRNA expression in dyspeptic patients. Microb Pathog.

[CR4] Baglan PH, Bozdayi G, Ozkan M, Ahmed K, Bozdayi AM, Ozden A (2006). Clarithromycin resistance prevalence and Icea gene status in *Helicobacter pylori* clinical isolates in Turkish patients with duodenal ulcer and functional dyspepsia. J Microbiol.

[CR5] Boyanova L, Yordanov D, Gergova G, Markovska R, Mitov I (2010). Association of iceA and babA genotypes in *Helicobacter pylori* strains with patient and strain characteristics. Antonie Van Leeuwenhoek.

[CR6] Brennan DE, Dowd C, O'Morain C, McNamara D, Smith SM (2018). Can bacterial virulence factors predict antibiotic resistant *Helicobacter pylori* infection?. World J Gastroenterol.

[CR7] Broutet N, Marais A, Lamouliatte H, de Mascarel A, Samoyeau R, Salamon R, Megraud F (2001). cagA Status and eradication treatment outcome of anti-*Helicobacter pylori* triple therapies in patients with nonulcer dyspepsia. J Clin Microbiol.

[CR8] Chang YW, Ko WJ, Oh CH, Park YM, Oh SJ, Moon JR, Cho J-H, Kim J-W, Jang JY (2019). Clarithromycin resistance and female gender affect *Helicobacter pylori* eradication failure in chronic gastritis. Korean J Intern Med.

[CR9] De Francesco V, Zullo A, Ierardi E, Giorgio F, Perna F, Hassan C, Morini S, Panella C, Vaira D (2010). Phenotypic and genotypic *Helicobacter pylori* clarithromycin resistance and therapeutic outcome: benefits and limits. J Antimicrob Chemother.

[CR10] Elviss NC, Owen RJ, Xerry J, Walker AM, Davies K (2004). *Helicobacter pylori* antibiotic resistance patterns and genotypes in adult dyspeptic patients from a regional population in North Wales. J Antimicrob Chemother.

[CR11] Fujikawa A, Shirasaka D, Yamamoto S, Ota H, Yahiro K, Fukada M, Shintani T, Wada A, Aoyama N, Noda M (2003). Mice deficient in protein tyrosine phosphatase receptor type Z are resistant to gastric ulcer induction by VacA of *Helicobacter pylori*. Nat Genet.

[CR12] Ghotaslou R, Leylabadlo HE, Asl YM (2015). Prevalence of antibiotic resistance in *Helicobacter pylori*: a recent literature review. World J Methodol.

[CR13] Godoy APO, Ribeiro ML, Benvengo YHB, Vitiello L, Miranda MCB, Mendonça S, Pedrazzoli J (2003). Analysis of antimicrobial susceptibility and virulence factors in *Helicobacter pylori* clinical isolates. BMC Gastroenterol.

[CR14] Goudarzi M, Heidary M, Azad M, Fazeli M, Goudarzi H (2016). Evaluation of antimicrobial susceptibility and integron carriage in *Helicobacter pylori* isolates from patients. Gastroenterol Hepatol Bed Bench.

[CR15] Hooi JKY, Lai WY, Ng WK, Suen MMY, Underwood FE, Tanyingoh D, Malfertheiner P, Graham DY, Wong VWS, Wu JCY, Chan FKL, Sung JJY, Kaplan GG, Ng SC (2017). Global prevalence of *Helicobacter pylori* infection: systematic review and meta-analysis. Gastroenterology.

[CR16] Ji Z, Han F, Meng F, Tu M, Yang N, Zhang J (2016). The association of age and antibiotic resistance of *Helicobacter pylori*: a study in Jiaxing City, Zhejiang Province, China. Medicine.

[CR17] Karabiber H, Selimoglu MA, Otlu B, Yildirim O, Ozer A (2014). Virulence factors and antibiotic resistance in children with *Helicobacter pylori* gastritis. J Pediatr Gastroenterol Nutr.

[CR18] Kargar M, Ghorbani-Dalini S, Doosti A, Souod N (2011) Real-time PCR assay using allele-specific TaqMan probe for detection of clarithromycin resistance and its point mutations in *Helicobacter pylori*. J Isfahan Med Sch. 29(126).

[CR19] Khademi F, Poursina F, Hosseini E, Akbari M, Safaei HG (2015). *Helicobacter pylori* in Iran: a systematic review on the antibiotic resistance. Iran J Basic Med Sci.

[CR20] Khaledi M, Bagheri N, Validi M, Zamanzad B, Afkhami H, Fathi J, Rahimian G, Gholipour A (2020). Determination of CagA EPIYA motif in *Helicobacter pylori* strains isolated from patients with digestive disorder. Heliyon.

[CR21] Khan A, Farooqui A, Manzoor H, Akhtar SS, Quraishy MS, Kazmi SU (2012). Antibiotic resistance and cagA gene correlation: a looming crisis of *Helicobacter pylori*. World J Gastroenterol.

[CR22] Korona-Glowniak I, Cichoz-Lach H, Siwiec R, Andrzejczuk S, Glowniak A, Matras P, Malm A (2019). Antibiotic resistance and genotypes of *Helicobacter pylori* strains in patients with gastroduodenal disease in Southeast Poland. J Clin Med.

[CR23] Lee I, Lee H, Kim M, Fukumoto M, Sawada S, Jakate S, Gould VE (2005). Ethnic difference of *Helicobacter pylori* gastritis: Korean and Japanese gastritis is characterized by male- and antrum-predominant acute foveolitis in comparison with American gastritis. World J Gastroenterol.

[CR24] Loivukene K, Kolk H, Maaroos HI, Kasenomm P, Ustav M, Mikelsaar M (2000). Metronidazole and clarithromycin susceptibility and the subtypes of vacA of *Helicobacter pylori* isolates in Estonia. Scand J Infect Dis.

[CR25] Malekzadeh R, Derakhshan MH, Malekzadeh Z (2009). Gastric cancer in Iran: epidemiology and risk factors. Arch Iran Med.

[CR26] Malekzadeh R, Setodeh R, Amini M, Vakili A, Ansari R, Massarat S (1997) Effect of furazolidone, bismuth subcitrate and amoxicillin on eradication of Helicobacter pylori (HP) in Iran*.* Paper presented at the Gastroenterology.

[CR27] Marshall BJ, Warren JR (1984). Unidentified curved bacilli in the stomach of patients with gastritis and peptic ulceration. Lancet.

[CR28] Mitui M, Patel A, Leos NK, Doern CD, Park JY (2014). Novel *Helicobacter pylori* sequencing test identifies high rate of clarithromycin resistance. J Pediatr Gastroenterol Nutr.

[CR29] Molina-Infante J, Romano M, Fernandez-Bermejo M, Federico A, Gravina AG, Pozzati L, Garcia-Abadia Elena, Vinagre-Rodriguez Gema, Martinez-Alcala Carmen, Hernandez-Alonso M (2013). Optimized nonbismuth quadruple therapies cure most patients with *Helicobacter pylori* infection in populations with high rates of antibiotic resistance. Gastroenterology.

[CR30] Montecucco C, de Bernard M (2003). Molecular and cellular mechanisms of action of the vacuolating cytotoxin (VacA) and neutrophil-activating protein (HP-NAP) virulence factors of *Helicobacter pylori*. Microbes Infect.

[CR31] Nahid-Samiei M, Rahimian G, Shafigh M, Taheri F, Karami-Hurestani M, Sanaei M-J, Heshmati M, Bagheri N (2020). Enhanced frequency of CD19+ IL-10+ B cells in human gastric mucosa infected by *Helicobacter pylori*. Am J Med Sci.

[CR32] Pandya H, Agravat HH, Patel J, Sodagar N (2014). Emerging antimicrobial resistance pattern of *Helicobacter pylori* in central Gujarat. Indian J Med Microbiol.

[CR33] Rhead JL, Letley DP, Mohammadi M, Hussein N, Mohagheghi MA, Eshagh Hosseini M, Atherton JC (2007). A new *Helicobacter pylori* vacuolating cytotoxin determinant, the intermediate region, is associated with gastric cancer. Gastroenterology.

[CR34] Roghani HS, Massarrat S, Pahlewanzadeh MR, Dashti M (1999). Effect of two different doses of metronidazole and tetracycline in bismuth triple therapy on eradication of *Helicobacter pylori* and its resistant strains. Eur J Gastroenterol Hepatol.

[CR35] Sanaei M-J, Shirzad H, Soltani A, Abdollahpour-Alitappeh M, Shafigh M-H, Rahimian G, Heshmati M, Bagheri N (2020). Up-regulated CCL18, CCL28 and CXCL13 expression is associated with the risk of gastritis and peptic ulcer disease in *Helicobacter pylori* infection. Am J Med Sci..

[CR36] Sanaii A, Shirzad H, Haghighian M, Rahimian G, Soltani A, Shafigh M, Tahmasbi K, Bagheri N (2019). Role of Th22 cells in *Helicobacter pylori*-related gastritis and peptic ulcer diseases. Mol Biol Rep.

[CR37] Sanches BS, Martins GM, Lima K, Cota B, Moretzsohn LD, Ribeiro LT, Breyer H-P, Maguilnik I, Maia A, Rezende-Filho J (2016). Detection of *Helicobacter pylori* resistance to clarithromycin and fluoroquinolones in Brazil: a national survey. World J Gastroenterol.

[CR38] Sugano K, Tack J, Kuipers EJ, Graham DY, El-Omar EM, Miura S, Haruma K, Asaka M, Uemura N, Malfertheiner P (2015). Kyoto global consensus report on *Helicobacter pylori* gastritis. Gut.

[CR39] Taneike I, Nami A, O'Connor A, Fitzgerald N, Murphy P, Qasim A, O’CONNOR, H., O'Morain, C. (2009). Analysis of drug resistance and virulence-factor genotype of Irish *Helicobacter pylori* strains: is there any relationship between resistance to metronidazole and cagA status?. Aliment Pharmacol Ther.

[CR40] Thung I, Aramin H, Vavinskaya V, Gupta S, Park JY, Crowe SE, Valasek MA (2016). Review article: the global emergence of *Helicobacter pylori* antibiotic resistance. Aliment Pharmacol Ther.

[CR41] Treiber G, Wittig J, Ammon S, Walker S, van Doorn LJ, Klotz U (2002). Clinical outcome and influencing factors of a new short-term quadruple therapy for *Helicobacter pylori* eradication: a randomized controlled trial (MACLOR study). Arch Intern Med.

[CR42] Wang D, Guo Q, Yuan Y, Gong Y (2019). The antibiotic resistance of *Helicobacter pylori* to five antibiotics and influencing factors in an area of China with a high risk of gastric cancer. BMC Microbiol.

[CR43] Zhang M (2015). High antibiotic resistance rate: a difficult issue for *Helicobacter pylori* eradication treatment. World J Gastroenterol.

